# *Burkholderia cepacia*, a cause of post pars plana vitrectomy silicone oil related endophthalmitis: clinico-pathological presentation and outcome of management

**DOI:** 10.1186/s40942-018-0138-7

**Published:** 2018-09-26

**Authors:** Ogugua Ndubuisi Okonkwo, Adekunle Olubola Hassan, Olufemi Oderinlo, Michael Ekuoba Gyasi

**Affiliations:** 1 Eye Foundation Retina Institute, 27 Isaac John Street, GRA, Ikeja, Lagos Nigeria; 2 St Thomas Eye Hospital, Accra, Ghana

**Keywords:** Endophthalmitis, Vitrectomy, Silicone oil, *Pseudomonas aeruginosa*, *Burkholderia cepacia*

## Abstract

**Aim:**

To report the long-term outcome of the management of a series of culture proven post pars plana vitrectomy endophthalmitis in which the infective agent was in the silicone oil used as an endotamponade. The isolates were *Burkholderia cepacia* and *Pseudomonas aeruginosa*.

**Method:**

A retrospective interventional reporting of a series consisting of a cluster of five cases.

**Cases:**

Five consecutive patients received the same batch of 5000-centistoke silicone oil as endotamponade at the conclusion of vitreoretinal surgery and presented with features of acute intraocular inflammation, which was due to an infective cause. The infective organism isolated from the mixture of silicone oil and fluid was *B. cepacia* in three out of the initial cluster of four eyes and *P. aeruginosa* in the fifth eye.

**Outcome of management:**

The initial 4 eyes evolved into eyes with poor vision (hand motion, perception of light and no perception of light), advanced proliferative vitreoretinopathy, hypotony, phthisis bulbi and cornea opacity. The poor visual outcome was deemed to be consequent to delay in removal of the silicone oil, despite use of intravitreal, systemic and topical antibiotics. The fifth case, because of the heightened index of suspicion gained from the preceding four cases, had a prompt removal of the silicone oil, vitreal lavage with antibiotics, and intravitreal injection of antibiotics and steroid. He regained a 6/9 vision.

**Conclusion:**

Gram-negative bacilli can colonize silicone oil resulting in post pars plana vitrectomy endophthalmitis. The index of suspicion for this should be high and can be managed successfully with prompt removal of the silicone oil, microbial sensitive antibiotic lavage of the vitreous cavity, followed by a repeat tamponade.

## Background

Silicone oil has had an important use as a long-acting endotamponade agent in vitreoretinal surgery for several decades [1]. It is used in particular following surgery for complex retina detachments such as in giant retina tear, trauma, viral retinitis, proliferative vitreoretinopathy (PVR) and chronicity [2]. Infective endophthalmitis is a dreaded severe complication of intraocular surgery due to intraocular infection by microbes. Silicone oil has been proposed to have antimicrobial properties and so endophthalmitis associated with its use is very rare [3–5]. In addition, due to the perceived inhibitory effect of silicone oil on microbes, its use as a tamponade post vitrectomy for infective endophthalmitis is often welcomed. This inhibitory effect of silicone oil on microbes has been demonstrated on the following common endophthalmitis causing microbes including *Staphylococcus aureus*, *Staphylococcus epidermidis*, *Escherichia coli*, *Pseudomonas aeruginosa*, and *Candida albicans*.

In the five cases we present, silicone oil was colonized by microbes and was the source of microbial contamination. Since silicone oil related endophthalmitis is rare, its presentation, treatment strategy and outcome of treatment is not well known.

We present the clinical course and treatment outcome in a series of five eyes in five consecutive patients who developed post pars plana vitrectomy endophthalmitis secondary to culture proven gram-negative bacilli. In these eyes, the infective organisms were *Burkholderia cepacia* in three of the first four eyes and *P. aeruginosa* in fifth and final case. The infectious agent in all five cases was inoculated into the eye through the silicone oil and is believed to have colonized the silicone oil. Silicone oil related endophthalmitis (SORE) is used to represent this clinical situation. Therefore, using these five cases we wish to point out the treatment strategy that resulted in an unfavorable outcome and the strategy, which resulted in improved visual outcome. There are few case reports of endophthalmitis in a silicone filled eye (SORE) as show in Table 1. Our series represents the largest number of eyes reporting this clinical condition.

**Table 1:** Previous reports of SORE including number of eyes

Authors	Year of publication	Number of cases/eyes
Chong et al. [6]	1986	1
Johnson et al. [7]	1989	2
Zimmer-Galler et al. [8]	1997	1
Goel et al. [9]	2015	1
Steinmetz et al. [10]	2018	2

## Case series

Five cases of SORE were seen in a little over a 1-year period and managed by the team at the department. All five eyes presented with complex or chronic retinal detachment. There were three males and two females. Three of the eyes were myopic, one was emmetropic and the refractive status of one eye was not known. All five cases received the same batch of 5000 centistoke (CS) silicone oil as tamponade after pars plana vitrectomy procedure to reattach the rhegmatogenous retinal detachment. All other vitreoretinal surgeries performed within the same period as the five eyes (e.g. for vitreous hemorrhage or macular holes), without use of the incriminating batch of silicone oil did not have this presentation.

There was an initial cluster of four cases; which had undergone vitrectomy surgery in mid 2011. One of the four patients had a re vitrectomy for retina re detachment and proliferative vitreoretinopathy (PVR), while one of the patients had a 360-degree encircling band to his surgery. The fifth case had surgery in 2012. Ethical approval was sought from the review board of the Eye Foundation, but this was waived as the study only involved the retrospective review of case records.

## Case presentations

### *Case 1*

A 60  year old female presented with a 2-year history of retinal detachment in the right eye. A right eye PVR inferior bullous retinal detachment involving the macular reduced the vision to hand motion. A chorioretina scar was present in the nasal mid peripheral retina, while a large retina break was noted in the temporal periphery at about 10 o’clock position. The Left eye was satisfactory at this time.

The patient had a right eye uneventful vitrectomy and silicone oil injection on the 29th of April 2011. The retina was entirely reattached under the silicone oil.

**Fig. 1:**
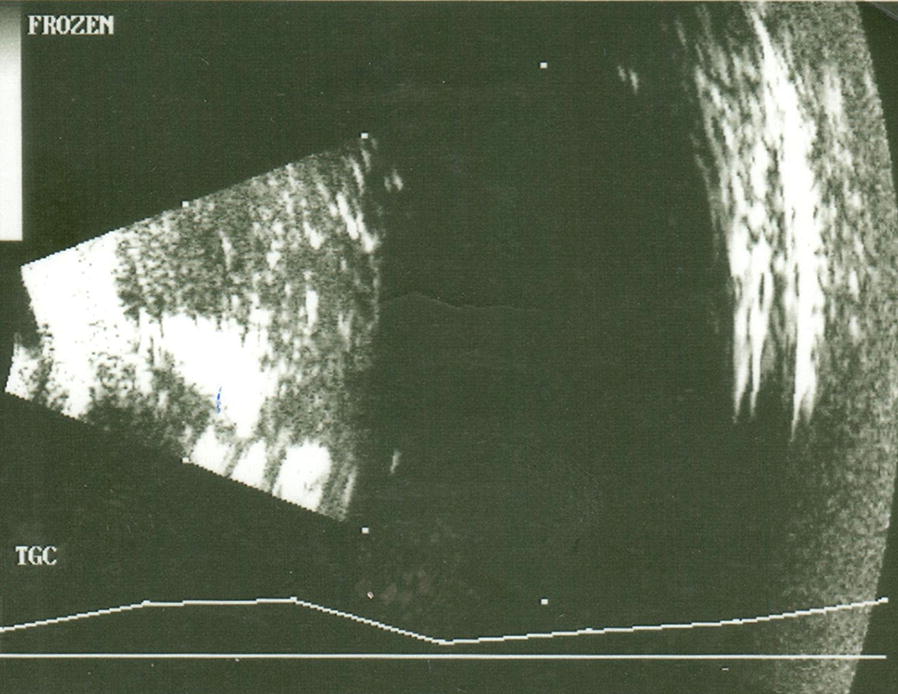
Case 1 B scan ultrasonography of the right eye done on the 11th of August before silicone oil removal, showing high echogenicity in the anterior vitreous cavity

Post operatively, by the first day through to the first month she received topical steroid and antibiotic preparations with the vision initially improving to 6/60. She however developed ptosis, erythema and significant keratic precipitates (KPs) and the silicone oil became opaque with an absence of the previously seen retina view. A diagnosis of ocular inflammation due to delayed onset post vitrectomy endophthalmitis was made (Fig. 1).

Silicone oil was removed on the 12th of August 2011.

Microbiology study: of the silicone oil was reported as;

“Gram stains could not be done, as the stains could not adhere to the silicone oil. Also no growth was seen after 40 h of culture”.

Hypopyon in the immediate postoperative stage was managed with frequent topical antibiotics and steroids (moxifloxacin and dexamethasone).

Cataract surgery was performed on the 4th of November 2011 facilitating view of the retina. A generalized PVR process occurred, vision deteriorating from hand motion to light perception and IOP of 2 mmHg. The inflammation was controlled with use of topical medications though a phthisical globe was the eventual outcome.

The left eye developed a para papillary choroidal neovascular membrane and intravitreal antiVEGF therapy was given.

### *Case 2*

A 34-year old female bilateral myope, presented with a 2-month history of right eye retinal detachment. A macular involving inferior retina detachment and multiple inferior retina breaks reduced the visual acuity to 6/60 + 1. The left eye had a prophylactic retina laser to peripheral retina breaks and lattice degeneration.

Uneventful right eye vitrectomy and silicone oil exchange were performed on the 1st of July 2011 with a fully reattached retina post operatively. Visual acuity was counting fingers on the first postoperative day and improved to 6/36 by the first week. The patient had significant ocular pain and conjunctiva hyperemia that were beyond normal expectation. The patient’s vision deteriorated to hand motion with complaints of increasing ocular pain and increased hyperemia. The patient developed a ptosis and the fundal view became hazy. The diagnosis was a post pars plana vitrectomy endophthalmitis.

The patient was initially treated with frequent topical moxifloxacin, dexamethasone, atropine and intravitreal injections of vancomycin and ceftazidime (as per the EVS protocol).

On the 9th of August 2011, the silicone oil was removed and cataract surgery was also performed. There was a hypoypon on post operative day 1.

**Fig. 2:**
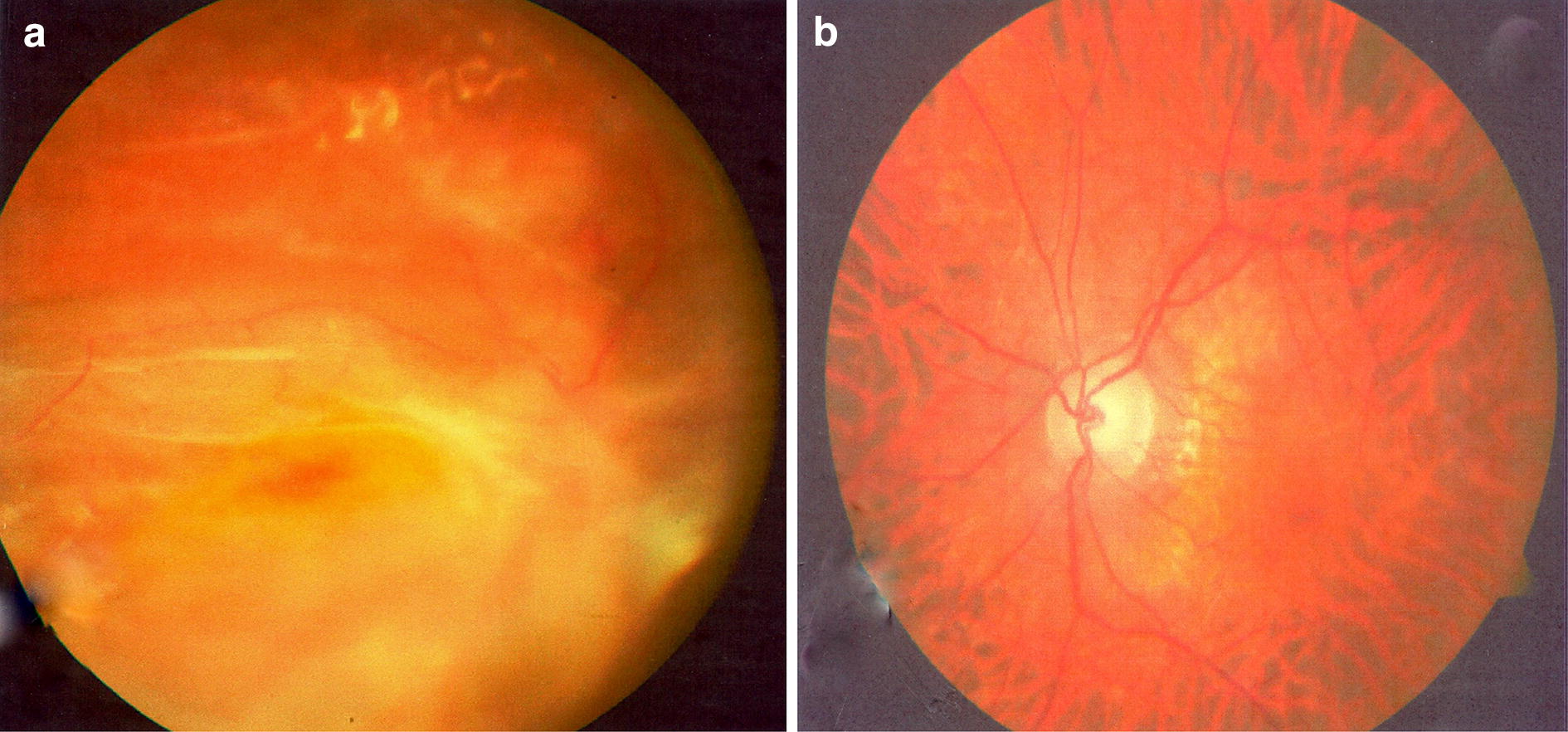
**a**, **b.** Shows the right eye with advance PVR process under the silicone oil and normal appearing left eye

The retina re-detached postoperatively and silicone oil was re-injected into the vitreous cavity on the 20th of August 2011 after further retina reattachment surgery (Fig. 2).

Microbiology study: of effluent from the vitreous cavity and silicone oil yielded; gram-negative bacilli and *Pseudomonas* species were isolated, sensitive to ceftazidime and co-trimoxazole, but resistant to amikacin, gentamicin and ciprofloxacin.

The patient was then placed on oral co-trimoxazole and continuous topical moxifloxacin and dexamethasone with complete resolution of the inflammation and severe PVR reducing vision in this eye to hand motion and IOP of 2 mmHg.

### *Case 3*

A 34-year old male bilateral high myope presented with a long-standing right eye retina detachment. Fundal examination revealed an inferior PVR macular involving retina detachment reducing the visual acuity to counting finger. The patient had multiple retina breaks in the inferior retina periphery and received prophylactic retina laser to the affected eye.

On the 8th of July 2011, the patient had a right eye combined vitrectomy with an encircling band and silicone oil injection. Post operatively the retina was reattached and the vision remained counting fingers. He was prescribed postoperative topical dexamethasone and moxifloxacin preparations. At the first post operative week there was considerable conjunctiva hyperemia and lid edema, visual acuity was noted to be hand motion.

Within the first month, he complained of significant tearing and vision had deteriorated to light perception. The eye was still hyperemic with significant chemosis, cornea opacity, hypopyon, an evolving cataract and posterior synechia. A corneal stromal abscess was noted during his subsequent visit. The diagnosis was an acute type post vitrectomy infectious endophthalmitis.

This was managed with frequent topical moxifloxacin and dexamethasone.

On the 31st of August 2011 a combined silicone oil removal and cataract surgery was performed.

Microbiology study: silicone oil mixed with fluid from the vitreous cavity yielded gram-negative bacilli and the organism isolated was Burkholderia (*Pseudomonas*) cepacia. It was sensitive to ceftazidime and co-trimoxazole, but resistant to ciprofloxacin, amikacin and gentamicin.

**Fig. 3:**
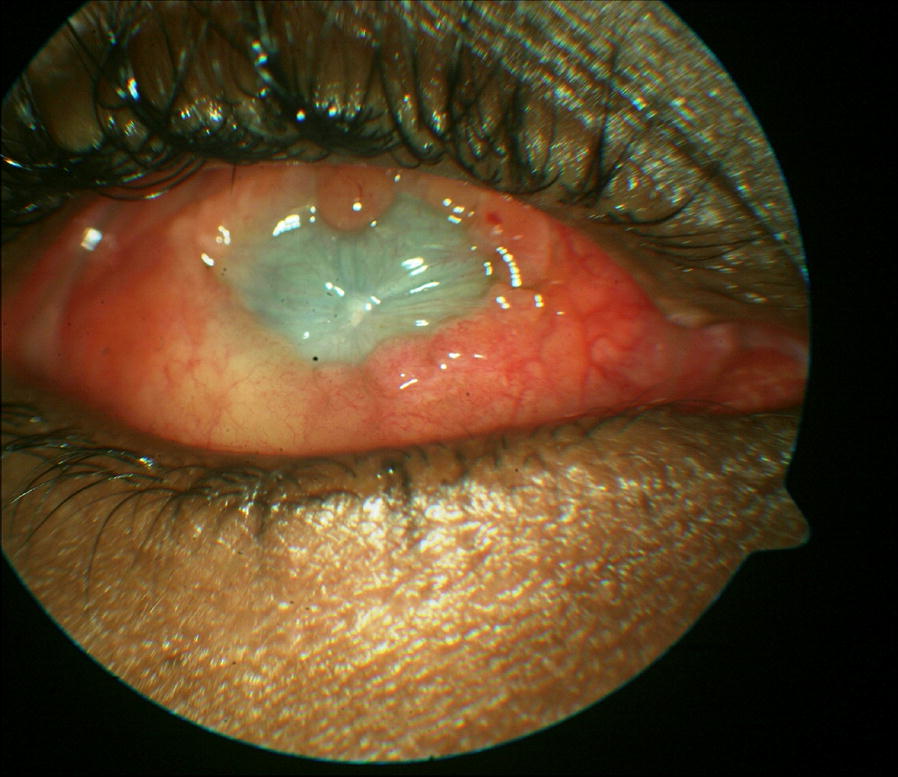
Case 3 treatment outcome results in a phthisical globe

Topical medications as before were continued. The vision deteriorated to no light perception, a phthisical globe and corneal opacity as shown on Fig. 3. The left eye remained normal.

### *Case 4*

A 43-year old male who had previously undergone a left eye vitrectomy with silicone oil and a 360-degree encirclement buckle for an inferior PVR retina detachment involving the macular, had reduced vision in the eye to 6/36. Surgery was performed on the 20th of May 2011. He suffered a post operative hyphema with bleeding into the silicone oil and vision reduced to hand motion in the post operative period. As the hemorrhage into the silicone oil did not clear. On the 8th of July 2011, the hemorrhagic silicone oil was removed and fresh silicone oil was re-injected.

Post operatively, the patient’s vision remained limited to hand motion. The patient developed ocular inflammation with symptoms of ptosis, lid swelling, hyperemia, chemosis, cornea ulcer and flare in the anterior chamber as in case three.

At the first post operative week there was significant hyperemia and by the subsequent visit the patient presented with a hypopyon. He  was placed on frequent topical moxifloxacin and dexamethasone. Intravitreal injections of vancomycin, ceftazidime (as per the EVS protocol) and dexamethasone were given. The patient went on to develop rubeosis, which was managed with intravitreal antiVEGF.

The silicone oil was removed on the 15th of September 2011.

Microbiology study: silicone oil mixed with vitreous effluent reported on microscopy numerous pus cells.

Bacterial culture: Burkholderia (*Pseudomonas* sp) cepacia, sensitive to ceftazidime and co trimoxazole but resistant to amikacin, ciprofloxacin and gentamicin.

He developed significant PVR with hypotony and a vision of hand motion.

### *Case 5*

**Fig. 4:**
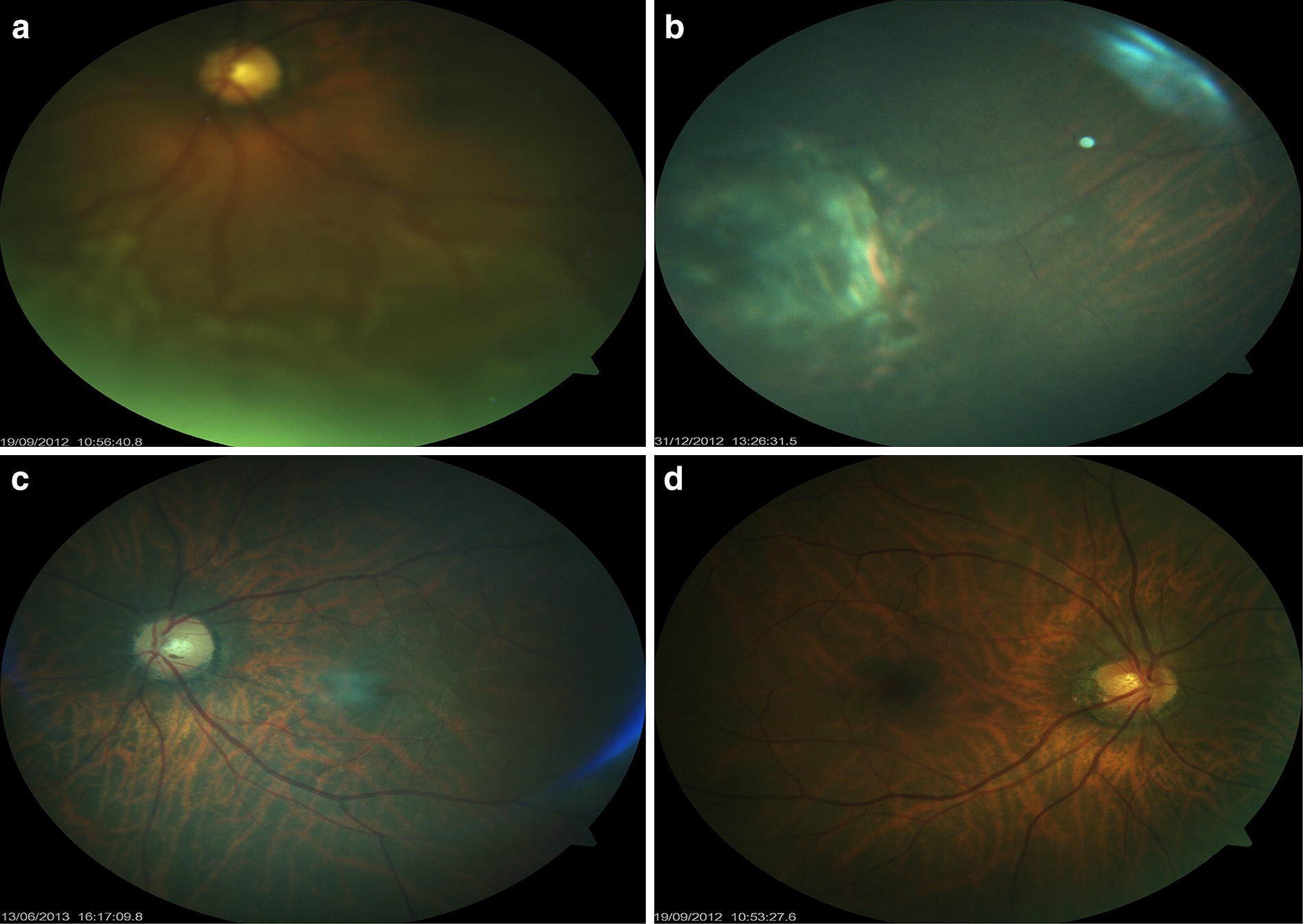
**a**–**d** Case 5 **a** an inferior retina detachment. **b** Laser induced chorioretina scar around the treated retina break. **c** Post operative attached retina appearance of the operated left eye. **d** Right eye

A 63-year old male who presented on the 18th of September 2012 with reduced left eye vision to 6/36 as a result of a 6-month period of retina detachment. Upon fundus examination an inferior macular involving chronic retinal detachment was observed (Fig. 4a). The patient was scheduled for a left eye vitrectomy and had an uneventful surgery on the 21st of September 2012. On the 1st post operative day his visual acuity was noted to be counting fingers with a reattached retina. At the next visit 1-week post surgery, he complained of pain in the left eye and vision remained limited to counting fingers but improved marginally to 6/60 with pinhole. At a subsequent visit he complained of reduced vision and foreign body sensation. His vision was reduced to hand motion, and he developed hyperemia and keratic precipitates. Posterior segment examination revealed opaque silicone oil. A faint retinal view could be seen. At his next visit few days later, there was severe conjunctival hyperemia, increasing number of anterior chamber cells and hypopyon. Due to this presentation and a heightened index of suspicion for SORE, the patient was scheduled for immediate silicone oil removal. On the 5th of October 2011, the silicone oil was removed. The procedure involved extraction of the silicone oil, multiple irrigation of the vitreous cavity with vancomycin and ceftazidime (in a concentration of 1 mg/0.1 ml and 2 mg/0.1 ml respectively) into a fluid filled eye. After this irrigation of the vitreal cavity, multiple air fluid exchange was performed. Then, 0.1 ml each of vancomycin, ceftazidime and dexamethasone was injected into the vitreous cavity. Intravitreal 10% C3F8 was used as tamponade after which the three sclerostomy sites were securely closed using 8–0 vicryl sutures.

Microbiology study: silicone oil and fluid mixture yielded heavy growth of gram-negative bacilli, which was isolated and identified as *P. aeruginosa*. It was sensitive to ceftazidime and co-trimoxazole, but resistant to amikacin, ciprofloxacin, levofloxacin, and gentamicin.

The patient was commenced on oral co-trimoxazole, frequent topical moxifloxacin and dexamethasone.

The intraocular inflammation settled completely with this treatment.

An uneventful left eye cataract surgery (phacoemulsification with implantation of posterior chamber intraocular lens) was performed on the 18th of December 2012. Post operatively, final vision settled to 6/9 with correction of − 0.50DS/− 3.00DC × 80. The patient’s eye remained quiet and retina attached with no PVR formation or macular edema (Fig. 4b, c). His fellow eye remains satisfactory (Fig. 4d).

A summary of the clinical presentation of SORE is seen in Table 2.

**Table 2:** Presenting symptoms and signs in five eyes of five patients with SORE

	Decreased vision	Pain	Conjunctival hyperemia	Upper eyelid swelling and/or Ptosis	Hypoyon	Keratic precipitates	Cornea opacity or abscess	Impaired fundal view and/or opaque silicone oil
Patient 1	Yes	No	Yes	Yes	Yes	Yes	No	Yes
Patient 2	Yes	Yes	Yes	Yes	Yes	Yes	No	Yes
Patient 3	Yes	No	Yes	Yes	Yes	Yes	Yes	Yes
Patient 4	Yes	No	Yes	Yes	Yes	Yes	Yes	Yes
Patient 5	Yes	Yes	Yes	No	Yes	Yes	No	Yes

Decrease in vision eventually occurred in all patients, including those with an initial immediate improvement in vision after vitrectomy surgery

## Treatment summary

In the four initial cases, treatment consisted of intravitreal antibiotics (vancomycin and ceftazidime using the EVS protocol with silicone oil insitu) and steroids (dexamethasone); oral antibiotics (moxifloxacin tablets); frequent topical antibiotics (moxifloxacin); steroids (dexamethasone); and cycloplegia (atropine). This did not appear to be an effective treatment strategy as eventual outcome was poor.

As the index of suspicion for SORE was low, initial diagnosis was that of a presumed sterile inflammatory process. The silicon oil was not removed until after a varied interval of time in the first four cases. The time between injection and removal of silicone oil in the first four scenarios ranged from 6 weeks to 3.5 months.

In the fifth case, because of the heightened index of suspicion, the silicone oil was removed after 2 weeks. Silicone oil removal was done as soon as symptoms and signs of ocular inflammation were noticed. At time of silicon oil removal multiple vitreal cavity lavage with antibiotic (vancomycin and ceftazidime as per EVS protocol) was injected into a fluid filled vitreous cavity and irrigated out using multiple air fluid exchange.

## Outcome of treatment

The outcome of treatment for the initial four cases was consistent with previously reported outcomes for post pars plana vitrectomy endophthalmitis. Most large series on post pars plana vitrectomy report significant visual loss. There are few reports of good visual outcomes however, with Mutoh et al. [11] reporting impressive outcomes of 20/20, 20/25 and 20/30 in three out of their four patients; this is similar to our fifth case that had a 6/9 vision.

Advanced PVR formation and hypotony also occurred in all four initial cases with eventual phthisis bulbi in two eyes.

## Discussion

Some of the lessons learnt from these five cases can be therefore summarized as follows;

*Burkholderia cepacia* though reported as a cause of endophthalmitis post cataract surgery, post trauma, and post intra vitreal antiVEGF has not previously been reported as a cause of post pars plana vitrectomy endophthalmitis. We report this series of cases in which *B. cepacia* was a cause of post pars plana vitrectomy endophthalmitis.

Though silicone oil has been known to exhibit antimicrobial activity against common causes of endophthalmitis; silicone oil does not appear to have a similar inhibitory effect on *B. cepacia* and perhaps not on all *Pseudomonas* sp., though this inhibitory effect has been previously reported to occur in relation to *P. aeruginosa*.

Treatment of silicone oil related endophthalmitis (SORE), when associated with *B. cepacia* can have extremely good results if there is prompt removal of the silicone oil, lavage of the vitreous cavity with sensitive antibiotics and repeat tamponade. A high index of suspicion for cases of SORE is required if a prompt diagnosis and treatment is to be achieved. A majority of the eyes however suffered extremely poor visual outcome when the silicone oil removal was delayed even with intravitreal injection of sensitive antibiotic (in this case ceftazidime) and use of frequent topical antibiotics and steroids.

*Burkholderia cepacia* is a non-fermentative, aerobic, gram-negative bacillus formerly classified as Pseudomonas. There is accumulated evidence to dispel earlier suggestions that the organism is avirulent and merely a marker of existing lung disease, as infection by the organism can result in devastating effects. However, unlike *P. aeruginosa*, *B. cepacia* is an organism of lower virulence with a limited ability to cause infection in humans [12].

*Burkholderia cepacia* previously known as *Pseudomonas cepacia* is one out of 17 species of the *B. cepacia* complex [13]. It was first discovered in the 1950s by Walter Hagemeyer Burkholder, an American plant pathologist, who observed that the organism had caused the soft rot of onion skin [13]. It was later renamed *B. cepacia* in the early 1990s.

It is found in various aquatic environments and is a frequent colonizer of fluids used in the hospital (e.g. irrigation solutions, intravenous fluids); it has been reported to colonize antiseptic solutions, hand wash and lotions [14] as well as even topical anesthetic eye drops [15]. *B. cepacia* is also known to survive and multiply in aqueous hospital environments, where it may persist for long periods [16]. *B. cepacia* rarely causes infection in healthy hosts. It is a common cause of lung infection in patients with cystic fibrosis, bronchiectasis and chronic granulomatous lung disease [16]. Ocular infection is rare, however there are case reports of *B. cepacia* ocular infection occurring as keratitis [17–19] and post cataract endophthalmitis [15, 20, 21]. In one such report there was a cluster of thirteen post cataract surgery endophthalmitis cases, which was due to *B. cepacia* colonizing the anesthetic solution [15].

This is the first report of *B. cepacia* causing post pars plana vitrectomy endophthalmitis. Furthermore, this is also the first report of this organism colonizing silicone oil; an endotamponade, which has previously been reported to have antimicrobial effect on *P. aeruginosa* [4]. In this report the silicone oil was identified to be the source of the infection because all the other pars plana vitrectomy cases done on the same day and within the same period as the five cases, but without use of this batch of silicone oil, did not have a similar endophthalmitis presentation. The surgeries of cases three and four were performed on the same day (8th of July 2011) and microbiology confirmed *B. cepacia* as the infective agent in both cases. All other vitrectomy cases done on the same day all followed a normal clinical course with no unusual inflammation. It is likely that case two, which was reported, as Pseudomonas species may have been *B. cepacia* as well.

In case one, which was the first in this series, the silicone oil sample extracted from the eye could not take up dyes for gram staining. The silicone oil sample ought to have been mixed with enough fluid from the eye for microscopy and culture; this was not done, as there was limited experience with this situation by then. Microbiology for case five was reported to be *P. aeruginosa*. It is not unlikely that *B. cepacia* and P. aeruginosa organisms may have co existed in this incriminating batch of silicone oil since co infection with the two microbes has been previously reported [17].

Post pars plana vitrectomy (ppv) endophthalmitis is rare and can present with a variable clinical course, the diagnosis can be delayed due to confusion from post operative inflammation, and a low index of suspicion. This was our experience in this case series. The rarity of this form of endophthalmitis makes the index of suspicion very low in a surgeon who has not previously experienced this scenario. Fortunately the incidence of ppv endophthalmitis is low and variable. It has been reported to be 0.07% [22], and 0.039% [23]. A larger series puts it at between 0.03 and 0.14% [24]. Risk factors for its occurrence include sutureless surgery, wound leakage, hypotony, and vitreous incarceration in the wound.

Microbiology: a good proportion may have a negative culture, however vitreous and anterior chamber tap may feature the following microbes including *S. aureus*, *Proteus mirabilus*, *S. epidermidis* and *P. aeruginosa*. Others include Propionibacterium, Enterococci, and Bacillus species. Most studies report that coagulase-negative Staphylococcus is the most common organism. Though *P. aeruginosa* appears on this list, *B. cepacia* does not.

For the five patients, the clinical presentation was with initial post operative visual acuity improvement in the immediate and early post operative stages, followed by a decline in vision to hand motion or light perception. Over a 1 week period all patients complained of increasing pain, and episodes of severe conjunctival injection despite being on routine post operative topical steroid and topical antibiotics. In one of the eyes there was prominence of keratic precipitates. Hypopyon was not a feature of this presentation in the early stages, but occurred later in almost all the patients. However vitreous opacity and early cataract development and progression was common. Table 2 gives a summary of the patient’s clinical presentation.

The silicone oil and other fluids extracted from all four initial cases investigated at two different laboratories grew colonies of *B. cepacia* and Pseudomonas sp. This was sensitive to ceftazidime and co-trimoxazole in all four cases, but resistant to amikacin, ciprofloxacin, gentamicin and levofloxacin. All patients had received oral ciprofloxacin 500 mg twice daily as part of routine post operative medications, but this was not effective based on resistant pattern reported to this antibiotic. The microbial sensitivity was similar to previous reports of *B. cepacia* endophthalmitis. The mechanism of *B. cepacia* resistance to commonly used antibiotics including aminoglycosides has been well described [25] and accounts for its multiple drug resistance.

To conclude, a suspicion of SORE should be entertained in a vitreoretina case in which silicone oil is used, if there is a decline of visual acuity after a short period of momentary improvement or stabilization, associated with increasing pain, severe hyperemia out of proportion with expectation, ptosis, hypopyon, cloudy silicone oil and perhaps cornea involvement. In such a case, therapy should involve immediate removal of the silicone oil and other treatments as earlier outlined. The specimen to be sent for microbiology should be a mixture of the silicone oil extracted as well as fluid from the vitreal cavity.

## Abbreviations

IOP: intraocular pressure
PVR: proliferative vitreoretinopathy
VEGF: vascular endothelial growth factor
EVS: endophthalmitis vitrectomy study
PPV: pars plana vitrectomy
